# MIR-491: CDKN2A tumor suppressor co-pilot

**DOI:** 10.18632/oncoscience.201

**Published:** 2015-08-21

**Authors:** Lynette M. Moore, Wei Zhang

**Affiliations:** Department of Pathology, The University of Texas MD Anderson Cancer Center, The University of Texas Graduate School of Biomedical Sciences, and ISB-MDA Genome Data Analysis Center, The Cancer Genome Atlas, Houston, Texas, USA

**Keywords:** miRNA, glioma

Genomic instability frequently results in changes in gene expression or altered regulation of oncogenes and tumor suppressor genes. Research into the tumorigenic impact of these genes has led to the current acceptance of many of these genes as drivers of tumorigenesis. In the past decade, studies of small, non-coding RNAs, such as microRNAs (miRNAs), have gained notice in the cancer research field. As miRNAs are often found in intragenic locations, it is likely that gene amplification and loss events such as those which disrupt known oncogenes and tumor suppressor genes, may also affect miRNAs. We recently reported that *MIR-491*, located just distal to the *CDKN2A* locus, is frequently co-deleted with *CDKN2A* [1]. As the *CDKN2A* locus encodes two well-studied and commonly altered tumor suppressors (p16^INK4A^ and p14^ARF^), we sought to determine whether loss of *MIR-491* plays a functional role in tumorigenesis or is a passenger event.

*MIR-491* encodes two mature miRNA; miR-491-3p and miR-491-5p, for which previous studies and *in silico* prediction tools identify several well-known oncogenes as targets. Our study aimed to determine whether *MIR-491* functions as a tumor suppressor gene in glioblastoma (GBM), by suppressing key cancer hallmarks through coordinate regulation of the predicted targets. We demonstrated that miR-491-5p directly targets *EGFR*, *CDK6*, and *Bcl-xL*; and that miR-491-3p directly targets *IGFBP2* and *CDK6*. Importantly, these predicted, and now confirmed targets of miR-491 each are major oncogenes in GBM. Re-expression of miR-491 impaired glioma cell proliferation and invasive capacity and impaired growth and self-renewal of glioma stem cells (GSCs). Ultimately, forced expression of miR-491-3p and -5p mimics in a murine GBM xenograft model resulted in prolonged survival. By targeting key oncogenes, the mature products of *MIR-491* coordinate effects on multiple hallmarks of cancer.

Co-deletion of *MIR-491* with *CDKN2A* also gives additional insight into observations surrounding major oncogenic contributors to GBM. Molecular alterations in EGFR have the potential to serve as therapeutic targets in GBM [2]. *EGFR* amplification occurs in 40-70% of primary GBM, yet some GBM tumors exhibit increased EGFR protein in the absence of gene amplification [3]. Deregulated miRNA, including miR-491, may impact EGFR overexpression. Another miR-491 target, *IGFBP2*, is one of the most consistently overexpressed factors in GBM, and is a demonstrated driver for tumorigenesis [4]. We previously identified an inverse relationship between IGFBP2 and p16^INK4A^ and p14^ARF^ in multiple cancer types [5]. However, at the time, a mechanistic understanding for this relationship was unknown. Identifying the co-deletion of *MIR-491* with *CDKN2A* provides key insight into this relationship. Re-expression of miR-491 in glioma cells and GSCs suppressed IGFBP2, and impaired many of the tumorigenic phenotypes (cell proliferation, invasion, and stem cell maintenance), which have been previously attributed, at least in part, to high levels of IGFBP2. Since our publication, several new studies have been published, solidifying the tumor suppressive role of this miRNA, confirming the direct targets of *MIR-491* we described, and identifying several new ones [6, 7]. Together, these studies demonstrate that *MIR-491* is a major tumor suppressor that coordinates a wide range of targets to impact key hallmarks of tumorigenesis.

Our observation that *MIR-491* is co-deleted with a major tumor suppressor locus, *CDKN2A*, indicates that disruption of normal miRNA expression patterns impacts tumorigenesis. MiRNAs can be located within gene loci, therefore genomic alterations which impact oncogenes and tumor suppressors may also deregulate miRNA. Additionally, these genomic alterations, if affecting miRNA processing pathway genes, may also affect miRNA expression patterns. Indeed, we recently reported that deregulation of the genes involved in processing miRNA is linked to glioma progression and patient survival and results in specific patterns of altered miRNA expression profiles [8].

**Figure 1:**
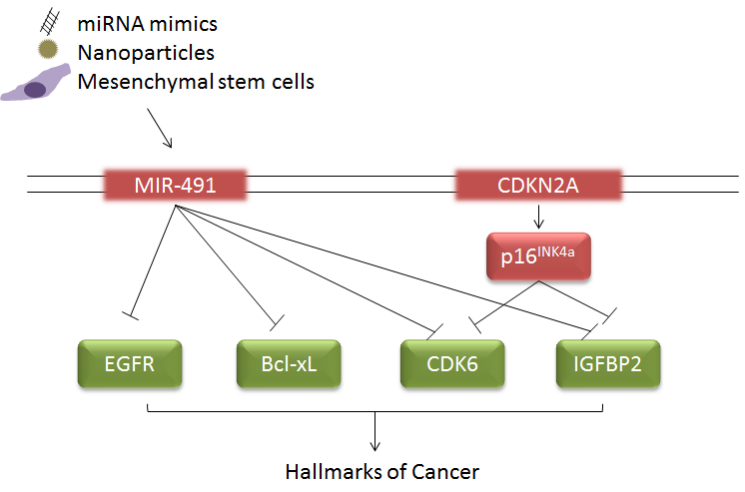
Co-deletion of *CDKN2A* and *MIR-491* relieves inhibition of oncogenic pathways and drives multiple hallmarks of tumorigenesis While therapeutic targeting of tumor suppressor gene loss is not a viable therapeutic strategy, tumor suppressive miRNA can be targeted by direct delivery of miRNA mimics or by using delivery methods such as nanoparticles or mesenchymal stem cells.

Due to their size and stability, miRNAs are becoming attractive potential new therapeutics. Several delivery mechanisms are being tested to target specific miRNAs to GBM and other tumor types. As *CKDN2A* loss itself is not directly druggable, the co-deletion of a tumor suppressive miRNA provides alternative options for therapeutics (Figure 1). Additionally, attempts to therapeutically inhibit the EGFR pathway in glioma have not achieved success, but delivery of miR-491 can impact both EGFR and other oncogenic pathways, perhaps achieving more successful results. As we have demonstrated, *MIR-491* loss is a frequent occurrence, not only in GBM, but in a wide range of other cancer types. Recent studies have also demonstrated the tumor-suppressive actions of miR-491 in many tumor types. With the development of new delivery mechanisms, miR-491 promises to be a novel, therapeutic strategy in many cancer types; and by targeting multiple cancer hallmarks, may also impair therapeutic resistance mechanisms of these tumors.
